# Supplementary material for: Evidence for unconventional superconductivity and nontrivial topology in PdTe
Source: Sci Rep. 2023 Apr 26;13:6824. doi: 10.1038/s41598-023-33237-5 (PMC10133450; doi:10.1038/s41598-023-33237-5)
Supplement: Supplementary file 1 — Supplementary Information. [file 41598_2023_33237_MOESM1_ESM.docx]

**Supplementary Material for Evidence for Unconventional Superconductivity and Nontrivial Topology in PdTe**

Ramakanta Chapai^1^, P. V. Sreenivasa Reddy^2^, Lingyi Xing^1^, David E. Graf^3^, Amar B. Karki^1^, Tay-Rong Chang^2^, and Rongying Jin^1,4^

^1^*Department of Physics and Astronomy, Louisiana State University, Baton Rouge, LA 70803, USA*

*^2^Department of Physics, National Cheng Kung University, Tainan 701, Taiwan*

^3^*National High Magnetic Field Laboratory, Tallahassee, FL 32310, USA*

*^4^Center for Experimental Nanoscale Physics, Department of Physics and Astronomy, University of South Carolina, Columbia, SC 29208, USA*

**Structure details:**

PdTe crystalizes in the NiAs-type hexagonal Bravais lattice with lattice parameters *a* = *b* = 4.1522 Å and *c* = 5.6712 Å with the space group *P6_3_/mmc* (No:194), as shown in Fig.S1(a). In this structure, each Pd atom is surrounded by six Te atoms and forms tilted octahedral local structures as shown in Fig.S1(b). The structure contains two formula units with four atoms in the unit cell [1].


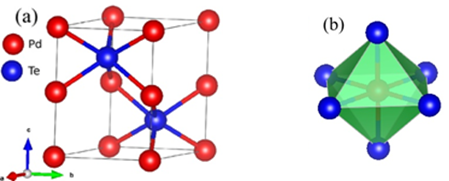


Fig. S1 (a) The crystal structure of PdTe. The red and blue spheres represent the Pd and Te atoms, respectively. (b) Octahedral local structure of PdTe.

As grown single crystals were screened for their orientiatons via x-ray diffraction (XRD) measurements in a PANalytical Empyrean x-ray diffractometer (Cu K_α_ radiation; λ = 1.54056 Å). The XRD pattern from a (00*l*) surface orientation is shown in Fig. S2(b). Few single crystals were crushed into fine powder and powder XRD measurement was performed. Fig. S2(a) shows the powder XRD pattern where all the diffraction peaks can be indexed under the NiAs-type hexagonal structure (space group *P*6_3_/*mmc*) with the lattice parameter *a* = *b* = 4.152(2) Å and *c* = 5.671(2) Å consistent with the previously reported values [1].


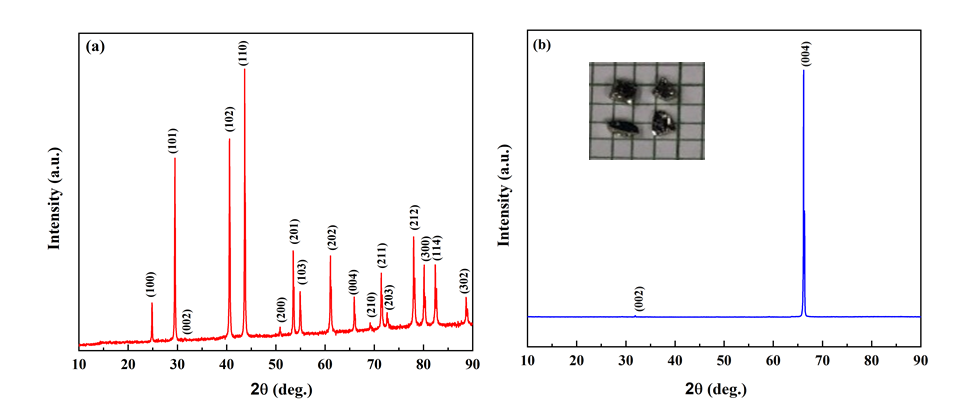


Fig. S2. (a) The powder X-ray diffraction (XRD) pattern of PdTe indexed in the NiAs-type hexagonal structure with space group P6_3_/mmc (194). (b) The XRD pattern of a single crystal of PdTe where the indexed peaks are from (*0 0 l*) plane. Inset: typical PdTe single crystals.

**Dingle plots:**

To get information about the dynamics of the carries, Dingle temperature (*T*_D_) is estimated through the Dingle plots. Figs. S3(a-d) present the Digle plots constructed for corresponding bands from the dHvA oscillations at *T* = 2 K for the case of *H*//*a*. From the slope of the linear fit in the Dingle plot *T*_D_ is obtained (displayed in the respective frame) which are listed on Table 1 in the main text.


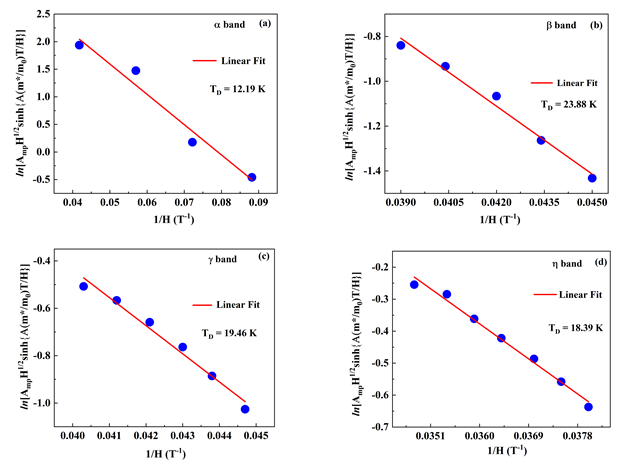


Fig. S3. The Dingle plots from dHvA oscillations in PdTe with *H*//*a* at 2 K for α, β, γ and η bands.

**Landau fan diagrams for γ and η bands:**

The dHvA oscillations at *T* = 2 K under *H*//*a* are used to construct the Landau fan diagrams. Suitable band-pass filtering [2] based on the frequency obtained via FFT analysis is applied to isolate the respective bands. Landau fan diagrams are then constructed by assigining the oscillation minima to *n*-1/4 and maxima to *n*+1/4, where *n* is the Landau level index [3]. The Landau fan diagram thus obtained for the γ and η bands are presented in Figs. S4 (a-b). Comparing the linear fits with the Lifshitz-Onsager quantization criterion, corresponding frequency and Berry phase are obtained which are listed in Table 1 in the main text.


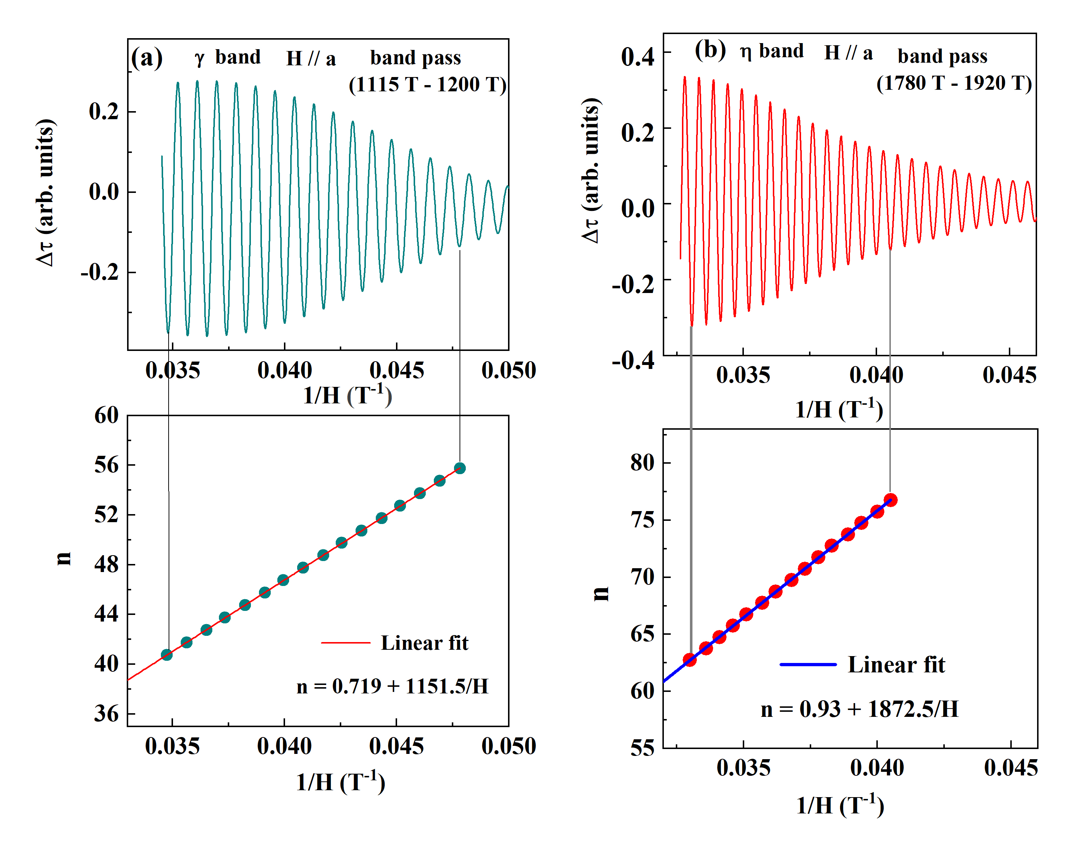


Fig. S4. Landau fan diagrams constructed from dHvA oscillation in magnetic torque of PdTe at 2 K under *H*//*a* for (a) γ band and (b) η band.

**Orbital projected band structure:**

To identify the topological nature of PdTe, we calculate the surface spectral weight throughout the (100) surface Brillouin zone using the semi-infinite Green’s function approach [4]. Fig. S5 shows the orbital projected band structure. We notice the topological Dirac surface state in the bulk gap forms α and β bands around the $\Gamma$ point, indicating that the α and β bands carry opposite parity eigenvalue. In addition, we observe the surface states that emerge out of the Dirac node that forming η and γ bands, suggesting the nontrivial topology of this Dirac state.


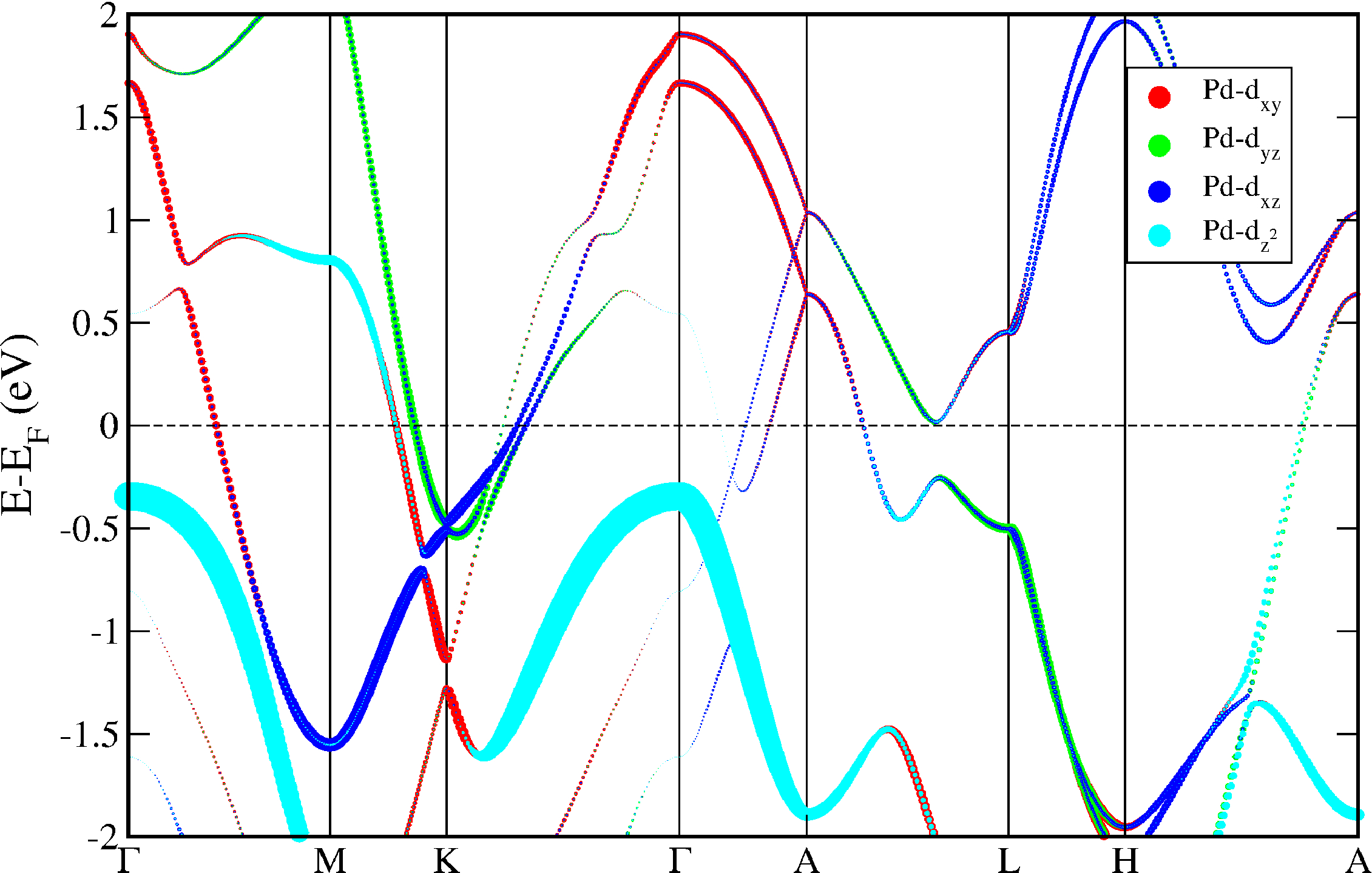

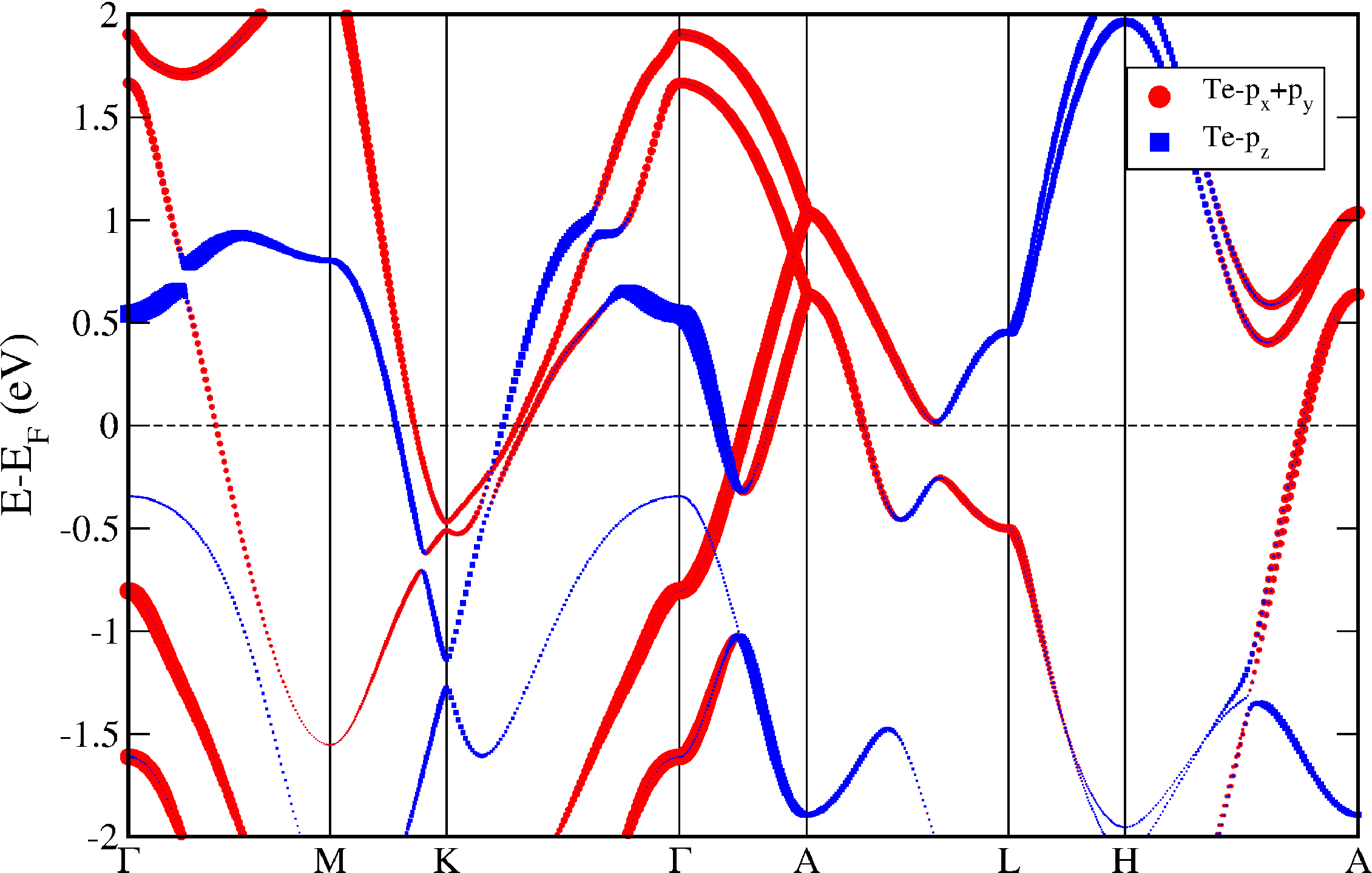


FIG. S5. Orbital projected band structure of PdTe with including spin orbit coupling.

**Surface band structures:**

**
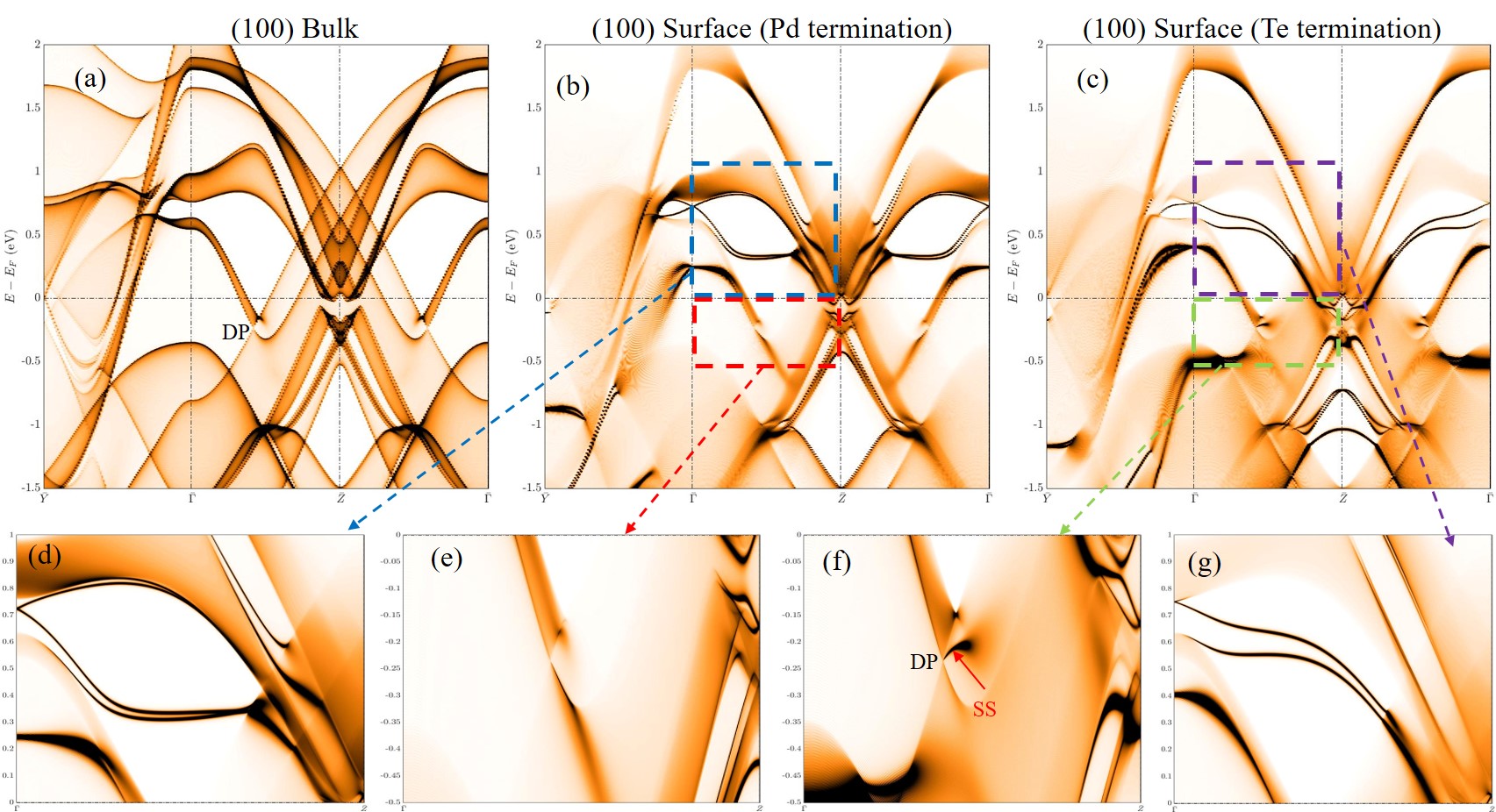
**

FIG. S6. Calculated (a) bulk, (b) Pd terminated surface and (c) Te terminated surface spectral weight for (100) surface of the BZ using semi-infinite Greens’s function. (d) and (e) are zoomed areas as highlighted in (b). (f) and (g) are zoomed areas as highlighted in (c).

**References**

**__________**

1. Karki, A. B., Browne, D. A., Stadler, S., Li, J. and Jin, R. PdTe: a strongly coupled superconductor. *J. Phys.: Condens. Matter* **24**, 055701 (2012).
2. Chapai, R. Browne, D. A., Graf, D. E., DiTusa, J. F. and Jin, R. Quantum oscillations with angular dependence in PdTe_2_ single crystals. *J. Phys.: Condens. Matter* **33**, 035601 (2021).
3. Shoenberg, D. *Magnetic Oscillations in Metals* (Cambridge University Press, Cambridge 2009).
4. Bryant, G. W. Surface states of ternary semiconductor alloys: Effect of alloys fluctuations in one-dimensional models with realistic atoms. *Phys. Rev. B* **31**, 5166 (1985).
